# Saving Beds and Budgets: Real-World Efficacy, Safety, and Pharmacoeconomics of Long-Acting Lipoglycopeptides (LALs) in a Day Hospital Setting

**DOI:** 10.3390/pathogens15070740

**Published:** 2026-07-14

**Authors:** Andrea Marino, Emmanuele Venanzi Rullo, Giuseppe Pipitone, Alessandro Giorgio Geremia, Andrea De Vito, Antonio Albanese, Nicholas Geremia, Alessandro Franzò, Federica Cosentino, Paolo Italia, Maria Elena Ciuppa, Andrea Buzzi, Laura Santoro, Chiara Iaria, Giordano Madeddu, Carmelo Iacobello, Rosa Manuele, Fabrizio Pulvirenti, Bruno Cacopardo, Giuseppe Nunnari

**Affiliations:** 1 Infectious Diseases Unit, Department of Clinical and Experimental Medicine, ARNAS Garibaldi Hospital, University of Catania, 91122 Catania, Italy; alefranzosr@gmail.com (A.F.); federicacosentino91@gmail.com (F.C.); ita.paolo@libero.it (P.I.); andrea.buzzi.db@gmail.com (A.B.); cacopard@unict.it (B.C.); giuseppe.nunnari1@unict.it (G.N.); 2 Unit of Infectious Diseases, Department of Clinical and Experimental Medicine, University of Messina, 98124 Messina, Italy; emmanuele.venanzirullo@unime.it (E.V.R.); laurasantoro94@gmail.com (L.S.); 3 Infectious Diseases Unit, ARNAS Civico-Di Cristina, 90127 Palermo, Italy; mariaelena.ciuppa@community.unipa.it (M.E.C.); iaria.chiara@gmail.com (C.I.); 4 Unit of Infectious Diseases, “G. Rodolico-S. Marco” University Hospital, University of Catania, 95121 Catania, Italy; alegere@hotmail.it (A.G.G.); rosimanuele@gmail.com (R.M.); 5 Unit of Infectious Diseases, Department of Medicine, Surgery and Pharmacy, University of Sassari, 07100 Sassari, Italy; andreadevitoaho@gmail.com (A.D.V.); giordano@uniss.it (G.M.); 6 Infectious Diseases Unit, Ospedale Papardo, 98124 Messina, Italy; antonioalbanese@icloud.com (A.A.); carmelo.iacobello@gmail.com (C.I.); 7 Unit of Infectious Diseases, Department of Clinical Medicine, Ospedale “dell’Angelo”, 30174 Venice, Italy; nicholas.geremia@aulss3.veneto.it; 8 Unit of Infectious Diseases, Department of Clinical Medicine, Ospedale Civile “S.S. Giovanni e Paolo”, 30174 Venice, Italy; 9 Unit of Infectious Diseases, PO Vittorio Emanuele, ASP Caltanissetta, 93012 Gela, Italy; f.pulvirenti@asp.cl.it

**Keywords:** long-acting lipoglycopeptides, dalbavancin, oritavancin, OPAT, day hospital, Gram-positive infections

## Abstract

Background: Long-acting lipoglycopeptides (LALs), including dalbavancin and oritavancin, may facilitate outpatient-oriented management of Gram-positive infections by reducing the need for prolonged hospitalization and daily intravenous therapy. However, real-world evidence on their use in day hospital pathways, particularly for complex off-label infections, remains limited. This study evaluated the clinical effectiveness, safety, and economic impact of LAL-based day hospital management, comparing in-label acute bacterial skin and skin-structure infections (ABSSSI) with complex off-label indications. Methods: This retrospective, multicenter observational study included 160 adult patients treated with dalbavancin and/or oritavancin in a day hospital setting across nine Italian centers between January 2020 and December 2025. Indications were classified as in-label ABSSSI or off-label infections, including osteomyelitis, prosthetic joint infection, spondylodiscitis, endocarditis, septic arthritis, and prosthetic cardiac infection. The primary endpoint was clinical success at final follow-up. Secondary endpoints included adverse drug events, readmissions, inflammatory marker response, and a budget impact cost-offset analysis based on avoided inpatient bed-days. Results: Overall, 54.4% of patients received LALs for off-label indications, mainly osteomyelitis, prosthetic joint infection, and spondylodiscitis. Clinical outcome was available for 159 patients, with an overall success rate of 86.8% (138/159). Success rates were 90.4% for in-label ABSSSI and 83.7% for off-label indications, with no statistically significant difference between groups (*p* = 0.247). In exploratory analyses, monotherapy was associated with lower failure odds; this most likely reflects confounding by indication and should not be interpreted as a treatment effect. Inflammatory markers significantly decreased from baseline to end of therapy. Adverse drug events were uncommon (3.3%), mild, and did not lead to treatment discontinuation. In a scenario-based cost-offset model (no matched inpatient comparator), the strategy corresponded to 1107–2214 avoided inpatient bed-days; estimated net savings ranged from approximate cost-neutrality under conservative assumptions (€11,976) to €676,176 under maximum assumptions, and one-way sensitivity analysis showed a possible net loss at low inpatient daily-cost values—underscoring that these are modeled rather than observed savings. Conclusions: In this uncontrolled, retrospective cohort, LAL-based day hospital management was feasible and associated with favorable observed clinical outcomes, an acceptable safety profile, and a potential for cost offset in selected patients with Gram-positive infections, including complex off-label indications. Because no matched inpatient or conventional OPAT comparator was included, these findings should be regarded as hypothesis-generating and cannot establish comparative efficacy, superiority, or actual cost savings.

## 1. Introduction

The management of serious bacterial infections traditionally necessitates prolonged hospitalization, primarily to ensure the administration of intravenous (IV) antimicrobial therapy. While effective, this model places significant strain on healthcare resources, contributing to bed shortages, higher direct medical costs, and an increased risk of hospital-acquired complications [1,2]. In response to these challenges, Outpatient Parenteral Antimicrobial Therapy (OPAT) and day hospital (DH) pathways have emerged as critical strategies to facilitate early discharge or completely avoid hospital admission [3] for stable patients, preserving frailty status and well-being [4,5].

Despite the advantages of OPAT, traditional IV regimens often remain logistically burdensome, typically requiring daily drug administration or the maintenance of long-term vascular access devices (e.g., PICC lines), which are associated with their own risks of thrombosis, mechanical failure, and catheter-related bloodstream infections (CLABSI) [5,6,7]. The development of long-acting lipoglycopeptides (LALs), such as dalbavancin and oritavancin, represents a paradigm shift in this landscape. Characterized by prolonged half-lives that allow for once-weekly or even single-dose regimens, these agents eliminate the need for daily visits and permanent vascular access, making them uniquely suited for the day hospital setting [8,9].

For approved ABSSSI indications, dalbavancin is administered as a single 1500 mg dose or as 1000 mg followed one week later by 500 mg, whereas oritavancin is given as a single 1200 mg dose. In deep-seated, off-label infections dosing is not standardized: dalbavancin is most frequently used as repeated 1500 mg doses at 1- to 2-week intervals, while repeated-dose oritavancin regimens for osteomyelitis are less well defined [8,9].

Currently, regulatory approval for LALs is largely limited to acute bacterial skin and skin structure infections (ABSSSI) [10]. However, the pharmacokinetic profile of these agents makes them highly attractive for “off-label” use in deep-seated, complex infections that require extended treatment durations, such as osteomyelitis, prosthetic joint infections (PJI), and spondylodiscitis [9,11]. While the efficacy of LALs in ABSSSI is well-established in randomized controlled trials, real-world evidence regarding their safety and effectiveness in complex, off-label indications remains limited to observational cohorts and case series [12,13]. Furthermore, while the clinical benefits are becoming clearer, the specific economic impact of adopting LAL strategies in a day-hospital model—specifically regarding the quantification of avoided bed-days and direct cost offsets—requires further validation in multicenter settings to confirm results observed in simulation-based economic models [14,15].

Unlike most published LAL cohorts, which are single-center, indication-specific, or focused primarily on ABSSSI, our study examines a large, multicenter day-hospital model applied across a wide spectrum of deep-seated and prosthetic infections. Furthermore, no previous work has integrated clinical outcomes with real-world hospitalization avoidance and economic impact; our study represents one of the larger multicenter day hospital cohorts to integrate clinical outcomes with hospitalization avoidance and an economic cost-offset analysis across a broad spectrum of infections, although we did not perform a formal systematic comparison with prior cohorts.

The primary objective of this retrospective, multicenter observational study was to evaluate the real-world clinical outcomes and safety profile of long-acting lipoglycopeptides in a diverse cohort of adult patients. We specifically aimed to compare the efficacy of LALs in on-label ABSSSI versus complex off-label indications. Additionally, we sought to determine the economic value of this therapeutic strategy by conducting a cost-offset analysis to estimate the net savings attributable to avoided inpatient hospitalizations.

## 2. Materials and Methods

### 2.1. Study Design and Population

This was a retrospective, multicenter, observational study including a consecutive cohort of 160 adult patients (≥18 years) who received at least one administration of a long-acting lipoglycopeptide (LAL)—either dalbavancin or oritavancin—in a day hospital (DH) setting between 1 January 2020 and 31 December 2025 at nine participating centers in six Italian cities (Catania, Sassari, Palermo, Messina, Venice, and Gela).

Inclusion criteria were: age ≥ 18 years; receipt of at least one LAL dose in a DH setting for a documented or suspected Gram-positive infection; and sufficient medical record documentation to ascertain treatment outcomes. Exclusion criteria were: age < 18 years or insufficient medical records to determine clinical outcome.

The decision to initiate LAL therapy in the day hospital setting was made by the treating-infectious-disease specialist at each center, generally for clinically stable patients with a documented or suspected Gram-positive infection requiring prolonged parenteral therapy in whom admission avoidance or early discharge was feasible; additional considerations included difficult venous access, expected poor adherence to daily OPAT, and patient preference. As a retrospective multicenter study, no single uniform institutional protocol governed these decisions, and eligibility thresholds may have varied across centers.

All data were collected from patient electronic medical records and compiled into a centralized, anonymized database. The study was conducted in accordance with the Declaration of Helsinki and was approved by Ethics Committee Catania 2 (protocol n° 101/CECT2). Given the retrospective, observational design, informed consent was waived in accordance with local regulations.

### 2.2. Data Collection and Definitions

Data were extracted from electronic medical records using a standardized case report form and entered into an anonymized database. Collected variables included demographics, comorbidities, and Charlson Comorbidity Index (CCI) [16]. Indications were categorized as ABSSSI, endocarditis, spondylodiscitis, osteomyelitis, prosthetic joint infection, prosthetic cardiac infection, septic arthritis, or other; for primary analyses, ABSSSI was classified as in-label and all other indications as off-label, prioritizing the primary deep-seated diagnosis in patients with multiple syndromes. Care pathway was classified as de novo (LAL initiated without prior hospitalization for the index infection) or step-down (LAL initiated after inpatient admission), recording prior inpatient IV days for step-down cases. Treatment strategy was categorized as monotherapy versus combination therapy (≥1 concomitant systemic antibiotic), and the number of LAL administrations was recorded. Microbiological status (culture-positive vs. culture-negative) and key safety laboratories were abstracted; additional diagnostic criteria and laboratory definitions are provided in the Supplementary Materials—Methods [16,17].

Repeat LAL dosing was directed by the treating clinician according to real-world practice and was neither protocolized nor systematically guided. Clinical and laboratory monitoring, including inflammatory markers, renal function, and complete blood count, followed routine practice at each day hospital administration and at end of therapy, with subsequent follow-up scheduled at the discretion of the treating physician; the absence of a uniform monitoring and follow-up schedule across centers is acknowledged as a limitation. Dalbavancin was administered as a median of 2 doses (IQR 1–2), with an initial dose of 1500 mg in the majority of patients (118/141) and, when repeated, most commonly at a weekly interval (median 7 days). Oritavancin was given predominantly as a single 1200 mg dose (18/19). Therapeutic drug monitoring was performed in only 8/160 patients (5.0%); accordingly, its interpretation is limited and exploratory.

### 2.3. Study Endpoints

The primary endpoint was clinical success, defined as clinical cure at final follow-up after completion of long-acting lipoglycopeptide (LAL) therapy, without need for additional antibiotics or unplanned surgery for the index infection. Clinical failure included persistence/worsening requiring additional antimicrobial therapy or unplanned surgery, relapse, or infection-related death. Secondary endpoints were adverse drug events, change in serum creatinine, 30- and 60-day readmissions, and a budget impact analysis of avoided inpatient bed-days. Additional endpoint definitions are provided in the Supplementary Materials—Methods.

### 2.4. Statistical Analysis

Categorical variables were compared using χ^2^ or Fisher’s exact tests and continuous variables using Mann–Whitney U or Wilcoxon signed-rank tests, as appropriate. Univariable logistic regression screened predictors of clinical failure. Given the low number of failures, multivariable analysis used Firth penalized logistic regression with parsimonious covariate selection. To further assess confounding by indication, we performed an inverse probability of treatment weighting (IPTW) sensitivity analysis comparing monotherapy vs. combination therapy. Analyses were conducted on available cases; two-sided *p* < 0.05 was considered statistically significant. Additional modeling details are provided in the Supplementary Materials—Methods.

### 2.5. Economic Analysis

We conducted a hospital/National Health Service budget impact (cost-offset) analysis among clinically cured patients, estimating direct costs of the LAL–day hospital pathway (drug acquisition plus DH administration) versus a counterfactual inpatient strategy. Avoided length of stay was modeled by indication using literature-informed assumptions and applied to an inpatient daily cost in the base case. Uncertainty in the counterfactual was explored using three scenarios varying the proportion of patients expected to require full inpatient admission, with one-way sensitivity analyses on key parameters. Full assumptions and calculations are reported in the Supplementary Materials—Methods.

## 3. Results

**Figure 1:**
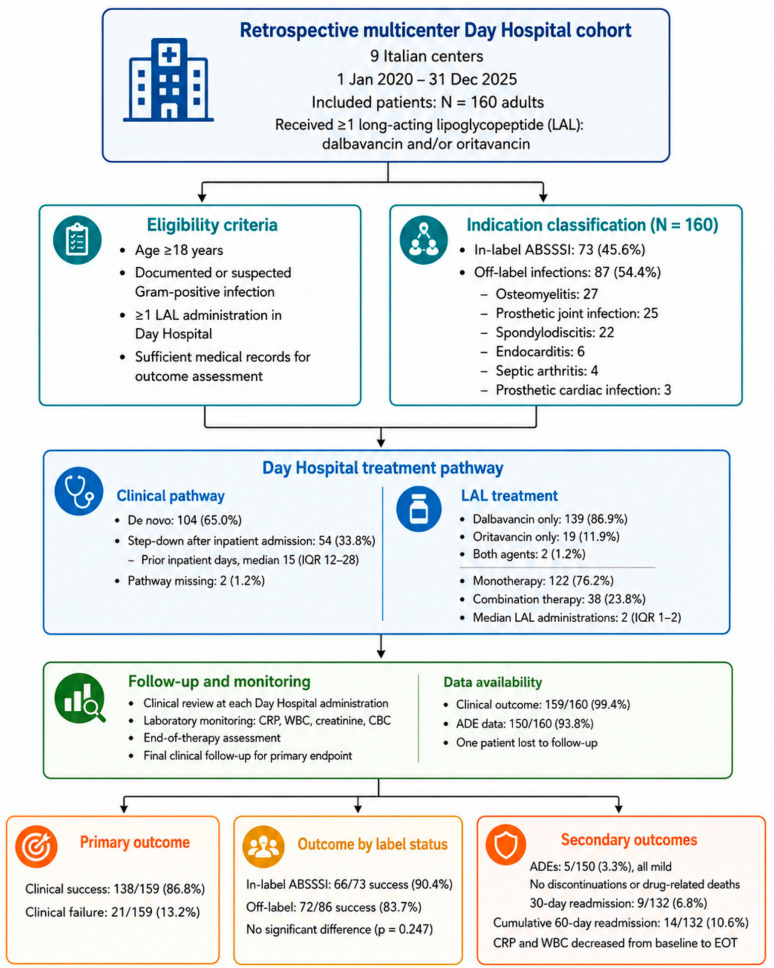
Study flow diagram.

Of 160 patients enrolled across nine centers, clinical outcome data were available for 159 (99.4%, one patient lost to follow-up). Adverse drug event data were available for 150 (93.8%) patients (Figure 1).

### 3.1. Patient Demographics and Baseline Characteristics

The cohort (*n* = 160) had median age 64 years and median CCI 3; 54.4% were treated for off-label infections, most commonly prosthetic joint infections and osteomyelitis (Table 1). LAL therapy was delivered through day hospital pathways with heterogeneous dosing consistent with real-world practice (Table 2).

### 3.2. Microbiology

Microbiological data were available for 144/160 patients (90%); 16 had no culture data recorded. A positive culture was obtained in 76/144 (52.8%) patients. Among culture-positive infections, MSSA was the most frequent pathogen (40/72, 55.6%), followed by MRSA (10/72, 13.9%). Coagulase-negative *staphylococci* were identified in 18/72 (25%) patients, including *S. epidermidis* (n = 10), *S. haemolyticus* (n = 5), *S. hominis* (n = 2), and *S. sciuri* (n = 1). *Enterococci* were identified in 2/72 (2.8%) patients. Four additional patients had positive cultures without species-level identification recorded. Sixty-eight patients (47.2% of those with culture data) had culture-negative infections treated empirically.

### 3.3. Prosthetic Device Data

Among the 160 patients, 43 had orthopedic prostheses and 10 had cardiac devices (pacemakers, valves, or vascular grafts); 1 patient had both. Prosthesis removal was performed in 23/52 (44.2%) patients: 21/43 (48.8%) orthopedic and 2/10 (20%) cardiac. Total explantation was performed in 19/23 (82.6%) and partial removal in 4 (17.4%).

### 3.4. Primary Efficacy Endpoint

Clinical follow-up data were available for 159 of 160 patients (99.4%). The overall clinical success rate was 86.8% (138/159; 95% CI 80.5–91.6%). Clinical failure was observed in 21/159 (13.2%) patients.

### 3.5. Secondary Efficacy Endpoints: Laboratory Response

LAL therapy was associated with a statistically significant reduction in inflammatory markers from baseline to EOT (Table 3). In the 121 patients with paired CRP data, the median CRP decreased from 5.40 mg/dL (IQR 1.00–14.89) to 1.50 mg/dL (IQR 0.60–4.90) (Hodges–Lehmann estimate −4.85; *p* < 0.001). In the 127 patients with paired WBC data, the median WBC count decreased from 7700 × 10^3^/μL (IQR 6250–10,960) to 7290 × 10^3^/μL (IQR 5535–8580) (Hodges–Lehmann estimate −1050; *p* < 0.001). Missingness analyses are reported in the Supplementary Materials—Results.

### 3.6. Safety and Tolerability

ADE data were available for 150/160 (93.8%) patients. ADEs were reported in 5/150 (3.3%) patients. All five ADEs were mild (Grade 1) and included pruritus (n = 3), allergic reaction (n = 1), and one episode of self-limiting nausea (n = 1). No serious ADEs, treatment discontinuations due to adverse events, or drug-related deaths were observed.

Paired creatinine data were available for 115 patients. Overall, the median creatinine did not change significantly: baseline 0.81 mg/dL (IQR 0.60–1.00) vs. EOT 0.80 mg/dL (IQR 0.65–1.00), Wilcoxon signed-rank test *p* = 0.388. When categorized by clinically significant changes: improved/stable (change < 0.3 mg/dL): 109/115 (94.8%); mild increase (0.3–0.5 mg/dL): 1/115 (0.9%); and moderate/severe increase (>0.5 mg/dL): 5/115 (4.3%). No patient required new dialysis. Among the five patients with a moderate/severe creatinine increase, the change was not clearly attributable to LAL therapy, and none required a change in LAL dosing or discontinuation of therapy; dalbavancin does not require dose adjustment for creatinine clearance ≥ 30 mL/min or in patients on regular haemodialysis.

### 3.7. Healthcare Utilization: 30-Day and 60-Day Readmission

The 30-day all-cause readmission rate was 9/132 (6.8%; 95% CI 3.2–12.5%). Among readmitted patients, 8 were readmitted for the index infection and 1 for an unrelated cause (pneumonia). The 31–60-day all-cause readmission rate was 5/126 (4.0%; 95% CI 1.3–9.0%), with all 5 occurring in patients not previously readmitted at 30 days. Cumulative 60-day readmission: 14/132 (10.6%).

### 3.8. Analysis of Clinical Failure and Risk Factors

In univariable logistic regression (Table 4), monotherapy was associated with lower odds of failure (OR 0.27, 95% CI 0.10–0.71; *p* = 0.008) and the number of LAL doses was positively associated (OR 1.41, 95% CI 1.04–1.92; *p* = 0.025). TDM showed a non-significant trend (OR 4.43, 95% CI 0.98–20.14; *p* = 0.054). None of the demographic, comorbidity, or baseline laboratory variables were significantly associated with failure.

Given 21 failures, multivariable modeling used parsimonious Firth regression (Table 5). Monotherapy was associated with lower odds of failure (aOR 0.25; 95% CI 0.09–0.71), while CCI was not significant. Because combination therapy was preferentially used in clinically more complex patients, this association was interpreted as confounding by indication, supported by baseline differences (Table 6) and a consistent IPTW sensitivity analysis (weighted OR 0.29; 95% CI 0.14–0.62). See the Supplementary Materials—Results for more information. These risk-factor analyses are exploratory and hypothesis-generating; the monotherapy–failure association reflects treatment-selection patterns rather than a causal effect. Monotherapy outcomes by infection type are summarized in Supplementary Table S1.

### 3.9. Subgroup Analysis: In-Label vs. Off-Label Indications

Despite differences in baseline complexity and treatment intensity between in-label and off-label groups, the clinical failure rate did not differ significantly between groups: off-label 16.3% (14/86) vs. in-label 9.6% (7/73) (Fisher’s exact *p* = 0.247). However, this comparison was underpowered and the non-significant result should not be interpreted as evidence of equivalent efficacy. Detailed outcomes by indication are shown in Table 7. Outcomes for the smallest subgroups—endocarditis (n = 5), prosthetic cardiac infection (n = 3), and septic arthritis (n = 4)—are descriptive only and too small to support inference about efficacy in these indications.

Clinical cure rates were similar between culture-positive (60/70, 85.7%) and culture-negative infections (56/63, 88.9%; *p* = 0.614), supporting the feasibility of empiric LAL use when microbiological confirmation is unavailable, although culture-positive patients had a significantly higher proportion of off-label indications (72.9% vs. 36.5%, *p* < 0.001).

### 3.10. Economic and Healthcare Resource Analysis

The cost-offset analysis included 138 cured patients with a total of 316 LAL administrations. Total LAL-DH pathway costs were €652,224 (drug acquisition: €604,824; DH visits: €47,400). Under the maximum counterfactual assumption (Scenario A, 100% full inpatient LOS), the strategy corresponded to 2214 avoided inpatient bed-days, primarily driven by ABSSSI and bone/joint infections (Table 8).

### 3.11. Base-Case Economic Results

Three scenarios were modeled reflecting uncertainty about the counterfactual (Table 9). Even under conservative assumptions (Scenario C, 50% of patients requiring full inpatient LOS), the LAL-DH pathway approached cost-neutrality (net savings €11,976; €87 per cured patient). Under realistic assumptions (Scenario B, 70%), savings reached €277,176 (€2009 per cured patient). Under maximum assumptions (Scenario A), savings amounted to €676,176 (€4900 per cured patient).

One-way sensitivity analyses around Scenario B are shown in Table 10. The model was most sensitive to assumed avoided LOS and inpatient daily cost. At a daily cost of €400 and 70% full LOS, the pathway yielded a net loss (−€32,624); at €800/day, savings rose to €586,976. The break-even point was 1087 avoided bed-days (49.1% of Scenario A), indicating cost-neutrality is achieved as long as approximately half of the patients would otherwise require full inpatient treatment.

### 3.12. Exploratory Secondary Economic Consequences

Assuming 4.9% HAI incidence per 10 inpatient days [18,19], the 1549 avoided bed-days (Scenario B) correspond to approximately 7.6 avoided hospital-acquired infections, yielding an additional €37,950 in cost avoidance at €5000 per HAI [20]. Among the 160 patients, 83 (51.9%) were of working age (<65 years), corresponding to approximately 804 avoided working-age bed-days. Including HAI avoidance, the total estimated economic benefit under Scenario B reaches approximately €315,000.

## 4. Discussion

Long-acting lipoglycopeptides (LALs) are increasingly used to deliver anti-Gram-positive therapy in outpatient-structured pathways because their long half-lives allow infrequent dosing and potentially reduce the need for prolonged inpatient care [18,19]. As shown by Gatti and colleagues in a comprehensive appraisal of dalbavancin beyond its approved indications, the growing real-world evidence base supports its use in prolonged-course infections such as osteomyelitis, prosthetic joint infection, endocarditis, and bloodstream infections, where traditional therapy often requires extended IV administration and healthcare contact [8]. In this context, our multicentre day hospital experience adds pragmatic evidence that LAL-based pathways can be implemented at scale across mixed indications with favorable clinical and organizational outcomes.

The heterogeneity of dosing schedules in our cohort reflects the interplay between the pharmacokinetics of long-acting lipoglycopeptides and the clinical context. For superficial ABSSSI, the prolonged half-life allows a single dose—or a two-dose regimen—to cover the entire treatment course, whereas deep-seated and prosthetic infections require sustained therapeutic exposure over weeks, prompting repeated dosing, typically at 1- to 2-week intervals, for the intended duration of therapy. In the absence of formal off-label dosing guidance, the number and interval of doses were individualized to infection type, severity, and clinical response.

As shown by observational series and pooled real-world experiences, dalbavancin has been repeatedly associated with high clinical success in heterogeneous Gram-positive infections when incorporated into early-discharge or outpatient pathways. For instance, Poliseno et al. reported that switching to dalbavancin in hospitalized patients with diverse Gram-positive infections shortened length of stay and treatment-related costs, and was associated with favorable clinical outcomes in difficult infections requiring prolonged therapy [20]. Our findings are consistent with this evidence, supporting the feasibility of DH-based management not only for ABSSSI but also for a substantial proportion of complex, off-label infections commonly encountered in routine care.

For oritavancin, the evidence base for off-label use is smaller but supports similar outpatient applicability in selected patients. As shown by Scoble et al. [9], real-world reports of oritavancin use in osteomyelitis suggest it can be an outpatient option with generally favorable clinical outcomes and limited adverse events, while acknowledging the need for further studies to define optimal dosing strategies in deep-seated infections. The consistency between our results and these published experiences supports the view that LAL-based DH pathways can offer a practical alternative when conventional prolonged IV therapy is difficult to deliver.

These findings should be interpreted in light of the differing strengths of evidence across indications. For ABSSSI, LAL efficacy is supported by randomized controlled trials, whereas for complex off-label deep-seated infections the evidence base remains observational, heterogeneous, and susceptible to selection bias. Our results should therefore be read as reinforcing feasibility in the off-label setting rather than as establishing efficacy comparable to that demonstrated for ABSSSI.

As shown across real-world LAL experiences, clinical failures in deep-seated infections are often concentrated in patients with greater baseline complexity, suboptimal source control, prosthetic material, or difficult-to-treat syndromes—features that also influence clinician choice of regimen [8]. For this reason, associations between treatment strategy (e.g., combination therapy) and clinical failure in retrospective cohorts should be interpreted primarily as markers of confounding by indication rather than causal effects. Our modeling strategy and interpretation align with this real-world framework: in routine practice, combination regimens are commonly selected when clinicians perceive a higher risk profile, and such selection can generate apparent associations with poorer outcomes even if the strategy itself is not harmful.

The off-label category in our study encompasses a clinically heterogeneous set of deep-seated infections—osteomyelitis, prosthetic joint infection, spondylodiscitis, endocarditis, septic arthritis, and prosthetic cardiac infection—that differ substantially in pathophysiology, the centrality of surgical source control, required treatment duration, and expected prognosis. Pooling them under a single “off-label” label is a simplification adopted to preserve analytic power, and the aggregate success rate should not be read as uniformly applicable across indications. Outcomes were least favorable in osteomyelitis (74.1%) and in the very small prosthetic cardiac infection subgroup, consistent with the recognized difficulty of achieving cure in these settings, whereas prosthetic joint infection and spondylodiscitis fared better. These indication-level differences argue for infection-specific evaluation rather than a single pooled off-label estimate.

The economic rationale for LAL pathways rests on the same mechanism emphasized in published economic evaluations: higher acquisition costs may be offset by reduced length of stay and avoided admissions. As shown by Marcellusi et al. [21] in a budget impact analysis of dalbavancin for ABSSSI in European health systems, pathway redesign with long-acting therapy can reduce inpatient resource use and yield favorable economic implications compared with standard antibiotic strategies. Similarly, Poliseno et al. [20] demonstrated that dalbavancin introduction was associated with reduced hospitalization and treatment-related costs, reinforcing that bed-day avoidance is a key driver of savings. Our cost-offset findings are directionally consistent with these evaluations and extend them to a multicentre DH implementation enriched with prolonged-course, off-label infections—where the opportunity for hospitalization avoidance is often greater than in ABSSSI-only cohorts.

It must be emphasized that these economic findings derive from a scenario-based budget-impact model built on assumed avoided length of stay and unit costs, not from observed, patient-level comparative costs against a real inpatient or OPAT control group. As the sensitivity analyses show, the direction and magnitude of the estimated savings depend heavily on these assumptions and can reverse to a net loss under conservative inpatient-cost inputs. The economic results should therefore be read as illustrative projections rather than demonstrated savings.

This study has several important limitations that qualify all of its conclusions. Foremost, its retrospective, single-arm observational design and the absence of a matched inpatient or conventional OPAT comparator preclude any inference about the comparative effectiveness, safety, or cost of LAL-based day hospital care relative to standard management; all outcomes reported here are descriptive rather than comparative.

As with other retrospective real-world analyses, our study is subject to residual confounding and clinician-driven selection of treatment pathways. Because eligibility for day hospital LAL treatment was determined locally, and the criteria and thresholds for this pathway likely differed across the nine participating centers, patients channeled into it were probably more clinically stable, more adherent, or better socially supported than the broader population with the same infections. This selection bias may inflate the apparent success rates, limits the generalizability of our findings, and is particularly relevant to the off-label indications, where the decision to use a LAL is more discretionary. In addition, our economic evaluation is a model-based cost-offset approach that relies on counterfactual assumptions about avoided inpatient length of stay and average unit costs, rather than patient-level micro-costing or a matched inpatient control cohort. This limitation is common to real-world budget impact modeling and should be kept in mind when interpreting absolute cost estimates. Finally, the evidence base for oritavancin in off-label deep-seated infections remains less mature than for dalbavancin; as highlighted in the osteomyelitis-focused review by Scoble et al., additional prospective work is needed to optimize dosing strategies and confirm comparative effectiveness across indications. Additional limitations warrant emphasis. A substantial proportion of infections were culture-negative; although Gram-positive etiology was inferred from a composite of clinical, radiological, inflammatory, and histopathological criteria, misclassification of the causative organism cannot be excluded. For the economic model, the step-down counterfactual is anchored to observed inpatient days, whereas the de novo counterfactual assumes an admission that would otherwise have occurred and is more speculative; the resulting estimates are therefore modeled projections rather than measured savings. Follow-up duration was variable and may underestimate late relapse in deep-seated infections. Finally, safety data were captured retrospectively without systematic active surveillance, and the observed adverse-event rate should be regarded as a lower bound.

Beyond individual clinical outcomes, our findings have important implications for healthcare system resilience. By shifting prolonged intravenous antimicrobial therapy from inpatient wards to structured day hospital pathways, LAL-based regimens can help mitigate chronic bed shortages, reduce exposure to hospital-acquired complications, and preserve inpatient capacity for higher-acuity conditions. The substantial number of avoided bed-days observed in our cohort demonstrates how these models can act as a buffer during periods of system stress—such as winter surges, increased surgical backlog, or infectious disease outbreaks—by enabling safe early discharge and preventing avoidable admissions. This reallocation of resources not only improves patient flow but also enhances operational flexibility, allowing hospitals to respond more effectively to fluctuating demand. In this sense, LAL-supported day-hospital models represent a pragmatic strategy for strengthening the adaptability and sustainability of modern healthcare systems.

## 5. Conclusions

As shown by published real-world and economic evaluations, LAL-based strategies can support outpatient-oriented pathways while maintaining high clinical effectiveness and potentially reducing inpatient resource utilization. Our multicentre day hospital experience is consistent with this literature and supports the feasibility of structured LAL pathways for both in-label ABSSSI and selected off-label deep-seated infections; however, as an uncontrolled observational study, it cannot demonstrate clinical equivalence or economic superiority relative to inpatient or conventional OPAT management, and broader integration should be guided by patient complexity and the need for source control.

## Figures and Tables

**Table 1:** Demographics and baseline comorbidities.

Characteristic	Value (N = 160)
Age (years)	
Median (IQR)	64.0 (51.0–74.5)
Sex, n (%)	
Male	103 (64.4%)
Female	57 (35.6%)
Charlson Comorbidity Index	
Median (IQR)	3.0 (1.0–5.0)
Individual Comorbidities, n (%)	
Hypertension	77 (48.1%)
Cardiac disease	55 (34.4%)
Diabetes mellitus	40 (25.0%)
Neurological disease	24 (15.0%)
COPD	21 (13.1%)
Active malignancy	15 (9.4%)

**Table 2:** Infection types and treatment details.

Characteristic	Value (N = 160)
Primary Indication, n (%)	
ABSSSI	73 (45.6%)
Osteomyelitis	27 (16.9%)
Prosthetic joint infections	25 (15.6%)
Spondylodiscitis	22 (13.8%)
Endocarditis	6 (3.8%)
Septic arthritis	4 (2.5%)
Prosthetic cardiac infection	3 (1.9%)
Label Status, n (%)	
In-label	73 (45.6%)
Off-label	87 (54.4%)
Clinical Pathway, n (%)	
De novo	104 (65.0%)
Step-down	54 (33.8%)
Missing	2 (1.2%)
Prior inpatient days (step-down), median (IQR)	15 (12–28)
LAL Agent, n (%)	
Dalbavancin only	139 (86.9%)
Oritavancin only	19 (11.9%)
Both agents	2 (1.2%)
Treatment Strategy, n (%)	
Monotherapy	122 (76.2%)
Combination therapy	38 (23.8%)
LAL Administrations, median (IQR)	2.0 (1.0–2.0)
TDM performed, n (%)	8 (5.0%)

**Table 3:** Inflammatory marker response (paired analysis).

Marker	n	Baseline Median (IQR)	EOT Median (IQR)	Hodges–Lehmann Estimate	*p*-Value
CRP (mg/dL)	121	5.40 (1.00–14.89)	1.50 (0.60–4.90)	−4.85	<0.001
WBC (×10^3^/μL)	127	7700 (6250–10,960)	7290 (5535–8580)	−1050	<0.001

**Table 4:** Univariable logistic regression for factors associated with clinical failure. The analysis included 159 patients with known clinical outcome (138 healed, 21 clinical failures).

Variable	OR (95% CI)	*p*-Value
Demographics and Comorbidities		
Male sex	1.93 (0.67–5.59)	0.223
Age (per year)	1.02 (0.99–1.05)	0.244
CCI (per point)	1.08 (0.93–1.25)	0.331
Type II diabetes mellitus	0.92 (0.31–2.69)	0.879
COPD	1.67 (0.50–5.57)	0.400
Hypertension	1.20 (0.48–3.01)	0.697
Coronary heart disease	1.50 (0.59–3.82)	0.395
Neurologic disorder	1.96 (0.64–5.97)	0.238
Active malignancy	1.11 (0.23–5.33)	0.901
Infection and Pathway		
Previous hospitalization (step-down)	2.01 (0.79–5.09)	0.141
ATB in previous hospitalization	2.00 (0.76–5.26)	0.160
Orthopedic prosthesis	0.81 (0.28–2.38)	0.707
Cardiac prosthesis	1.70 (0.34–8.60)	0.523
Treatment		
Monotherapy (vs. combination)	0.27 (0.10–0.71)	0.008
Number of LAL doses (per dose)	1.41 (1.04–1.92)	0.025
TDM performed	4.43 (0.98–20.14)	0.054
Dalbavancin use	2.81 (0.35–22.30)	0.328
Oritavancin use	0.70 (0.15–3.27)	0.652
Baseline Laboratory		
Hemoglobin (per g/dL)	0.86 (0.69–1.07)	0.174
CRP (per mg/dL)	1.00 (0.99–1.01)	0.817
Creatinine (per mg/dL)	1.27 (0.35–4.64)	0.713

**Table 5:** Firth penalized logistic regression: factors associated with clinical failure.

Variable	aOR (95% CI)	*p*-Value
Primary model (Firth penalized; EPV = 10.5)		
Monotherapy (vs. combination)	0.25 (0.09–0.71)	0.009
CCI (per point)	1.09 (0.93–1.28)	0.300
Sensitivity model (Firth penalized; EPV = 5.25)		
Monotherapy (vs. combination)	0.25 (0.09–0.72)	0.010
CCI (per point)	1.08 (0.92–1.28)	0.342
Off-label indication	0.99 (0.33–2.99)	0.991
Step-Down pathway	1.84 (0.65–5.23)	0.252

**Table 6:** Baseline characteristics by treatment strategy (confounding by indication assessment).

Characteristic	Monotherapy (n = 122)	Combination (n = 38)	*p*-Value
CCI, median (IQR)	3.0 (1.0–5.0)	3.0 (1.0–5.0)	0.473
Off-Label indication, n (%)	61/122 (50.0%)	26/38 (68.4%)	0.071
Step-Down pathway, n (%)	42/120 (35.0%)	12/38 (31.6%)	0.848
Prosthetic material, n (%)	36/122 (29.5%)	16/38 (42.1%)	0.212

**Table 7:** Clinical outcomes by infection type.

Indication	n	Cure	Failure	Success Rate (95% CI)
ABSSSI	73	66	7	90.4% (81.2–96.1)
Prosthetic joint infection	25	22	3	88.0% (68.8–97.5)
Spondylodiscitis	22	20	2	90.9% (70.8–98.9)
Osteomyelitis	27	20	7	74.1% (53.7–88.9)
Endocarditis	5	4	1	80.0% (28.4–99.5)
Prosthetic cardiac infection	3	2	1	66.7% (9.4–99.2)
Septic arthritis	4	4	0	100% (39.8–100)

**Table 8:** Avoided inpatient bed-days by indication (Scenario A: 100% full LOS).

Indication	Cured Patients (n)	Assumed LOS per Patient (Days)	Total Avoided Bed-Days
ABSSSI	66	10	660
Prosthetic joint infection	22	21	462
Spondylodiscitis	20	21	420
Osteomyelitis	20	21	420
Endocarditis	4	28	112
Prosthetic cardiac infection	2	28	56
Septic arthritis	4	21	84
Total	138	—	2214

**Table 9:** Budget impact analysis: three scenarios.

Scenario	Proportion Full LOS	Avoided Bed-Days	Counterfactual Inpatient Cost	LAL-DH Cost	Net Savings
A: Maximum	100%	2214	€1,328,400	€652,224	€676,176
B: Realistic	70%	1549	€929,400	€652,224	€277,176
C: Conservative	50%	1107	€664,200	€652,224	€11,976

**Table 10:** One-way sensitivity analyses (base case: Scenario B).

Parameter Varied	Range Tested	Net Savings Range
Inpatient daily cost	€400–€800	−€32,624 to €586,976
DH visit cost	€100–€200	€261,376–€292,976
Avoided LOS (±30%)	1084–2013 bed-days	−€1824 to €555,576
Include failure costs (21 × €6000)	+€126,000 to LAL costs	€151,176

## Data Availability

The data presented in this study are available on request from the corresponding author due to legal and ethical reasons.

## Supplementary Materials

The following supporting information can be downloaded at https://www.mdpi.com/article/10.3390/pathogens15070740/s1, Supplementary Materials—Methods, Supplementary Materials—Results.
