# Supplementary material for: Saving Beds and Budgets: Real-World Efficacy, Safety, and Pharmacoeconomics of Long-Acting Lipoglycopeptides (LALs) in a Day Hospital Setting
Source: Pathogens. 2026 Jul 14;15(7):740. doi: 10.3390/pathogens15070740 (PMC13414768; doi:10.3390/pathogens15070740)
Supplement: Supplementary file 1 [file pathogens-15-00740-s001.zip › Supplementary methods.pdf]

Supplementary Methods

Data collection and definitions

Baseline and follow-up data were extracted for each patient using a standardized case report form. Demographics and baseline characteristics included patient age, sex, and comorbidities (diabetes mellitus, COPD, hypertension, cardiac disease, neurological disease, active malignancy). The Charlson Comorbidity Index (CCI) was used to quantify the overall comorbidity burden [1].

The primary indication for LAL therapy was categorized as follows: ABSSSI, endocarditis, spondylodiscitis, osteomyelitis (non-spondylodiscitis), prosthetic joint infection (PJI), prosthetic cardiac infection (PCI), septic arthritis, or other. A primary analysis group, "Label Status," was created by classifying patients with ABSSSI as "In-Label" (n=73) and all other patients as "Off-Label" (n=87). Patients with dual diagnoses were classified according to the primary deep-seated diagnosis. For culture-negative infections, diagnosis was established using a combination of clinical presentation, radiological findings (e.g., MRI evidence of osteomyelitis or abscess formation), inflammatory markers, and surgical/histopathological findings when available. The decision to use empiric LAL therapy in culture-negative cases was made by the treating infectious disease specialist based on clinical likelihood of Gram-positive etiology.

Patients were stratified based on their care pathway: "De Novo" (LAL initiated in an outpatient setting without prior inpatient admission for the index infection) or "Step-Down" (LAL initiated as a continuation of therapy following a prior inpatient admission). For Step-Down patients, the duration of prior inpatient IV antimicrobial therapy was recorded. Treatment regimens were classified as "Monotherapy" (LAL only) or "Combination Therapy" (LAL administered with at least one other systemic antibiotic). The total number of LAL administrations was also recorded.

Laboratory markers, including C-reactive protein (CRP, mg/dL), white blood cell (WBC) count ($\times 10^3/\mu L$), hemoglobin (Hb, g/dL), platelets, procalcitonin (PCT), AST, ALT, and serum creatinine (mg/dL), were collected at baseline and at the end of treatment (EOT). The primary pathogen, when identified by culture or molecular methods, was recorded and categorized according to species. Methicillin resistance was determined by oxacillin susceptibility testing. Infections without microbiological confirmation were classified as culture-negative. For patients with prosthetic infections, whether prosthesis removal (partial or total) was performed was recorded.

Study Endpoints

The primary endpoint was clinical success, defined as clinical cure at the final follow-up assessment after completion of LAL therapy. Clinical cure was defined as resolution of signs and symptoms of infection (including normalization or substantial improvement of local signs, systemic symptoms, and inflammatory markers) without the need for additional antibiotic therapy or surgical intervention for the index infection at the time of EOT assessment. Clinical failure was defined as: persistence or worsening of infection signs/symptoms at EOT, need for alternative or additional antimicrobial therapy for the index infection during or immediately after LAL treatment, need for unplanned surgical intervention related to persistent infection, infection-related death, or documented microbiological or clinical relapse within the follow-up period.

Secondary efficacy endpoints included objective laboratory response (changes in CRP and WBC from baseline to EOT) and the need for additional antibiotic therapy after completion of the LAL regimen. Safety and tolerability endpoints included the incidence and nature of documented adverse drug events (ADEs) and the change in serum creatinine from baseline to EOT. Healthcare utilization endpoints included rates of 30-day and 60-day all-cause hospital readmission (calculated from the date of the last LAL administration) and the proportion of readmissions attributable to the index infection versus other causes.

Statistical analyses

All statistical analyses were performed using Python (SciPy 1.17, statsmodels) and IBM SPSS Statistics (Version 31.0). Two-sided p-values <0.05 were considered statistically significant for the primary endpoint and pre-specified secondary endpoints. For exploratory subgroup and risk factor analyses, results are described as hypothesis-generating, and nominal p-values are reported without adjustment for multiplicity.

The primary analysis included 159 of 160 patients with known clinical outcome. Continuous variables are presented as medians with interquartile range (IQR) or means with standard deviation (SD) as appropriate. Categorical variables are presented as frequencies and percentages. The 95% exact binomial (Clopper–Pearson) confidence interval (CI) was calculated for the primary endpoint.

Comparative analyses used chi-square test or Fisher's exact test for categorical comparisons, Mann–Whitney U test for comparisons of continuous variables between independent groups, and Wilcoxon signed-rank test for paired before-and-after data (baseline vs. EOT). Effect sizes for significant paired

comparisons are reported as Hodges–Lehmann estimates of median difference. No formal imputation was performed; analyses were conducted on available cases for each endpoint. To assess potential bias, we compared baseline characteristics between patients with and without paired laboratory data.

Univariable logistic regression was performed for all candidate predictors of clinical failure, reporting odds ratios (OR) with 95% confidence intervals. A multivariable logistic regression model (Model A) was then constructed including baseline characteristics (age, sex, CCI), infection-related variables (admission category, microorganism, sample type), and treatment-related covariates (monotherapy, number of doses, TDM, dalbavancin use, oritavancin use). At most one baseline laboratory covariate selected from univariable screening ($p<0.10$) was eligible for inclusion to limit overfitting; no baseline laboratory covariate met this threshold. Results are reported as adjusted odds ratios (aOR) with 95% CI. To better characterize potential confounding by indication, we created a descriptive table comparing baseline characteristics between patients who received combination therapy versus monotherapy.

Clinical outcomes were compared between In-Label (ABSSSI) and Off-Label indications. Renal safety was analyzed overall and stratified by clinically significant creatinine changes.

Economic Analysis

A cost-offset (budget impact) analysis was conducted from the hospital/National Healthcare Service perspective. This is not a cost-effectiveness analysis (CEA) and does not incorporate QALYs; rather, it estimates direct cost savings from avoided inpatient days. The analysis included all 138 clinically cured patients. Costs for the LAL-DH strategy were modeled as: (i) LAL drug acquisition costs plus (ii) DH administration costs. Drug acquisition cost was calculated per patient based on the actual number of administrations received, using a unit cost of €1,914 per 1,500 mg dose (reflecting the average acquisition price across participating centers). The DH visit cost was set at €150 per visit.

The counterfactual comparator was modeled as inpatient costs calculated as: inpatient daily cost × avoided length of stay (LOS). The base-case inpatient daily cost was set at €600, a conservative value commonly applied in Italian pharmacoeconomic analyses [2]. Avoided inpatient LOS was estimated by indication based on literature-informed assumptions: 10 days for ABSSSI (in-label), 21 days for off-label osteoarticular infections (PJI, spondylodiscitis, osteomyelitis, septic arthritis), and 28 days for endocarditis/cardiac device infections. For Step-Down patients who had already completed a portion of their anticipated inpatient stay, the counterfactual LOS was calculated as the total expected LOS minus the actual inpatient days already served.

To assess the robustness of the budget impact estimates, one-way sensitivity analyses were performed by varying key unit-cost parameters and avoided LOS assumptions within clinically plausible ranges: inpatient daily cost (€400–€800), DH visit cost (€100–€200), avoided LOS (±30% from base case), and inclusion of failure-related costs (estimated at €6,000 per failure event for readmission and additional therapy).

References

1. Charlson, M.E.; Pompei, P.; Ales, K.L.; MacKenzie, C.R. A New Method of Classifying Prognostic Comorbidity in Longitudinal Studies: Development and Validation. *J Chronic Dis* **1987**, *40*, 373–383, doi:10.1016/0021-9681(87)90171-8.
2. Foglia, E.; Ferrario, L.; Schettini, F.; Pagani, M.B.; Dalla Bona, M.; Porazzi, E. COVID-19 and Hospital Management Costs: The Italian Experience. *BMC Health Serv Res* **2022**, *22*, 991, doi:10.1186/s12913-022-08365-9.
