# Supplementary material for: Saving Beds and Budgets: Real-World Efficacy, Safety, and Pharmacoeconomics of Long-Acting Lipoglycopeptides (LALs) in a Day Hospital Setting
Source: Pathogens. 2026 Jul 14;15(7):740. doi: 10.3390/pathogens15070740 (PMC13414768; doi:10.3390/pathogens15070740)
Supplement: Supplementary file 1 [file pathogens-15-00740-s001.zip › Supplementary results.pdf]

Supplementary results

Laboratory response sensitivity analyses

A sensitivity analysis comparing baseline characteristics of patients with available paired CRP data (n=121) versus those without (n=39) showed no significant differences in sex (p=0.357), label status (p=0.794), or clinical pathway (p=0.060), though a trend toward higher CCI in the paired group was noted (p=0.094), suggesting that laboratory data were largely missing at random rather than systematically biased.

Analysis of clinical failure and risk factors

Given only 21 failure events, multivariable modeling was deliberately restricted to minimize overfitting. In the primary Firth penalized logistic regression model (2 covariates; EPV=10.5; Table 4), monotherapy remained associated with lower failure odds (aOR 0.25, 95% CI 0.09–0.71; p=0.009), while CCI was not significant (aOR 1.09, 95% CI 0.93–1.28; p=0.300). A sensitivity model adding off-label indication and Step-Down pathway (EPV=5.25) yielded consistent results (monotherapy aOR 0.25, 95% CI 0.09–0.72; p=0.010). These models are exploratory; confidence intervals and point estimates should be interpreted with caution given the low event count.

The apparent protective effect of monotherapy is best interpreted as evidence of selection bias. As shown in Table 5, clinicians selected combination regimens more often for patients with off-label indications (68.4% vs. 50.0%, p=0.071) and those with prosthetic material (42.1% vs. 29.5%, p=0.212), consistent with confounding by indication.

An IPTW analysis was performed to evaluate confounding further. After weighting, covariate balance improved substantially for the off-label indication (SMD reduced from 0.44 to 0.07), with acceptable balance across other covariates (all weighted SMD < 0.13). In the IPTW-weighted analysis, the monotherapy association persisted (IPTW-adjusted OR 0.29, 95% CI 0.14–0.62; p=0.001). However, because the propensity score model could include only measured covariates and could not capture unmeasured factors that drive treatment selection in clinical practice—such as acute infection severity, adequacy of source control, biofilm involvement, and the treating physician's clinical judgment—this result does not exclude residual confounding. We therefore present the monotherapy–failure association as primarily reflecting treatment selection patterns rather than as evidence of a causal effect of combination therapy on outcomes.

Indication	Evaluable (n)	Cured, n	Failure, n	Success rate
ABSSSI	61	56	5	91.8%
Prosthetic joint infection	17	14	3	82.4%
Spondylodiscitis	14	13	1	92.9%
Osteomyelitis	20	18	2	90.0%
Endocarditis	4	4	0	100%
Prosthetic cardiac infection	2	1	1	50.0%
Septic arthritis	3	3	0	100%
**Total**	**121**	**109**	**12**	**90.1%**

Table S1 - Cure and failure among monotherapy patients stratified by infection type. Outcomes are shown for the 121 evaluable monotherapy patients. One endocarditis patient treated with monotherapy was lost to follow-up and is excluded. Counts are consistent with the overall outcomes reported in Table 6.
